# New/Different Look at the Presence of *Aspergillus* in Mycobacterial Pulmonary Diseases. Long-Term Retrospective Cohort Study

**DOI:** 10.3390/microorganisms9020270

**Published:** 2021-01-28

**Authors:** Kiyoharu Fukushima, Hiroshi Kida

**Affiliations:** 1 Department of Respiratory Medicine, National Hospital Organization, Osaka Toneyama Medical Centre, 5-1-1 Toneyama, Toyonaka, Osaka 560-8552, Japan; kida.hiroshi.sv@mail.hosp.go.jp; 2 Department of Respiratory Medicine and Clinical Immunology, Osaka University Graduate School of Medicine, 2-2 Yamadaoka, Suita, Osaka 565-0871, Japan

**Keywords:** *Mycobacterium avium* complex, nodular bronchiectasis, non-tuberculous mycobacteria, pulmonary aspergillosis, *Asperugillus fumigatus*

## Abstract

Chronic pulmonary aspergillosis (CPA) has been reported to be associated with poor prognosis in non-tuberculous mycobacteria (NTM)-pulmonary disease (PD) patients. However, whether isolation of *Apergillus* species is associated with poor outcome or mostly just the reflection of colonization is a widely debated issue and a yet unsolved question. We conducted this single-centered retrospective cohort study of 409 NTM-PD patients to assess the impacts and prevalence of *Aspergillus* isolation and CPA development. The median observation time was 85 months. *Aspergillus* species were isolated from 79 (19.3%) and 23 (5.6%) developed CPA. Isolation of *Aspergillus* species was not associated with mortality in NTM-PD patients (*p* = 0.9016). Multivariate logistic regression analysis revealed that higher CRP (*p* = 0.0213) and AFB stain positivity (*p* = 0.0101) were independently associated with *Aspergillus* isolation. Different mycobacterial species were not associated with *Aspergillus* isolation. Survival curves for patients with CPA diagnosis were significantly and strikingly different from those without (*p* = 0.0064), suggesting that CPA development severely affects clinical outcome. Multivariate logistic regression analysis revealed that the use of systemic steroids (*p* = 0.0189) and cavity (*p* = 0.0207) were independent risk factors for the progression to CPA. Considering the high mortality rate of CPA in NTM-PD, early diagnosis and treatment are essential to improve outcomes for NTM-PD patients.

## 1. Introduction

Non-tuberculous mycobacteria (NTM) and *Aspergillus* species are ubiquitous organisms that cause various disorders in humans [1]. Previous studies have demonstrated that chronic pulmonary aspergillosis (CPA), a progressive debilitating disease often resulting in a shortened lifespan, was associated with poor prognosis in patients with NTM-pulmonary disease (PD) [2,3]. CPA is a semi-invasive form of pulmonary aspergillosis and classified into three distinct forms: Chronic cavitary pulmonary aspergillosis, chronic fibrosing pulmonary aspergillosis, and subacute invasive pulmonary aspergillosis (formerly chronic necrotizing pulmonary aspergillosis). Growing evidence suggested the close relationships between NTM and *Aspergillus* infection [4]. However, still now, the precise mechanisms of causal relationships among NTM and *Aspergillus* infection in the lung are not sufficiently understood. Broad spectrum multi-drug combination antibiotics are usually used for the treatment of NTM-PD, and this may increases the risk of fungal colonization including *Aspergillus* species because of selective pressure, in addition to airway clearance defects in bronchiectatic NTM-PD patients. This may predispose NTM-PD patients to *Aspergillus* lung infection particularly in patients with underlying lung structural change such as cavity, or weakened immunological state. On the other hand, regarding the diagnosis of CPA, previous studies are based on different diagnostic definitions in *Aspergillus*-related infectious conditions, and clinical and definite diagnosis of CPA. Clinical diagnosis of CPA is based on inflammatory immunological reactions caused by *Aspergillus* species; isolation of the organism itself is not absolutely necessary as negative culture results for *Aspergillus* species occur in about half of CPA patients [5,6]. Hence, clinical CPA consiss of heterogeneous populations of sero-positive and/or culture positive patients. Moreover, isolation of *Aspergillus* species from culture isolates of respiratory specimen often reflects colonization, and clinical significance and prognostic importance of *Aspergillus* isolation in NTM-PD patients is not well understood. Because NTM and *Aspergillus* are ubiquitous environmental organisms, difficulties exists in differentiation of colonization from active infection. Hence, attribution of causality of infection by one of these organisms is challenging. Therefore, whether isolation of *Aspergillus* species from respiratory tracts of NTM-PD patients was casually associated with poor outcomes or simply a reflection of colonization remains a widely debated and yet unsolved question [1,7]. We conducted this retrospective study, using the largest cohort and the longest observation period to date, to assess the significance of *Aspergillus* species isolated from respiratory specimens in NTM-PD patients, and to understand the similarities and differences between colonization and infection using definite diagnostic criteria for CPA. This is the first study to assess the impacts and prevalence of *Aspergillus* isolation and CPA among patients with NTM-PD using definite diagnostic criteria for CPA.

## 2. Materials and Methods

### 2.1. Study Design and Patients

This retrospective study was approved by the ethics board of the National Hospital Organization, Osaka Toneyama Medical Center (TNH-2019063). The medical records of patients with NTM-PD seen at the National Hospital Organization Osaka Toneyama Medical Center between January 2008 and October 2019 were retrospectively reviewed. Patients were included if they were ≥20 years old, met the American Thoracic Society (ATS)/Infectious Diseases Society of America criteria for NTM-PD [8], and underwent fungal cultures of respiratory specimen at least once during study period. Patients with simple aspergilloma were excluded. All patients were followed until their last visit, death, or the end of the study period (13 August 2020).

### 2.2. Data Collection

Baseline clinical parameters, including age, sex, body mass index (BMI), chronic obstructive pulmonary disease (COPD), old tuberculosis (Old Tb), use of systemic steroids, C-reactive protein (CRP), radiologic features, NTM species, and acid fast bacilli (AFB) stain positivity were obtained at the time of diagnosis of NTM-PD. Treatment duration, antimicrobial treatment for NTM-PD, bacterial culture results, and chest computed tomography findings were obtained from medical records.

### 2.3. Diagnosis of Chronic Pulmonary Aspergillosis (CPA)

Diagnosis of CPA was defined by fulfilment of all of these criteria; compatible clinical symptoms, compatible radiological findings, *Aspergillus* serology [positive Aspergillus IgG (Bio-Rad, Hercules, CA, USA), positive precipitins test (Microgen Bioproducts Ltd., Camberley, Surrey, UK), strongly positive Aspergillus antigen (Bio-Rad)], and isolation of *Aspergillus* species from respiratory samples. Simple aspergilloma (SA) was defined as a single fungal ball in a single pulmonary cavity, and evidence of Aspergillus infection. Each criterion for CPA diagnosis was based on the European Respiratory Society and European Society of Clinical Microbiology and Infectious Diseases guidelines for CPA [5]. *Aspergillus* colonization was defined as isolation of *Aspergillus* species from respiratory samples without evidence of chronic Aspergillus infection. Use of systemic steroids was defined as doses of more than 5 mg/day. Chest computed tomography (CT) findings were assessed by two pulmonologists blinded to the clinical data. Discrepancies were resolved through a consensus review by pulmonary physicians and a pulmonary radiologist. Diagnosis of CPA, SA, and *Aspergillus* colonization were made by two pulmonologists based on clinical data and chest CT findings.

### 2.4. Radiological Evaluation 

Radiographic abnormalities were classified according to distinct disease patterns observed on chest CT. Patients with fibrocavitary lesions and pleural thickening mainly in the upper lobes on CT were diagnosed with fibrocavitary (FC) disease, and patients with multiple nodules on CT and bronchiectasis were diagnosed with nodular bronchiectatic (NB) disease. Patients with no specific pattern on CT, such as solitary pulmonary nodules, were diagnosed with unclassifiable disease.

### 2.5. Sputum Examination

Sputum cultures were assessed for acid-fast bacilli using 2% Ogawa egg medium (Japan BCG, Tokyo, Japan) or mycobacteria growth indicator tubes (Becton, Dickinson and Company, Tokyo, Japan). Non-tuberculous mycobacterial species were identified using AccuProbe (Gen-Probe Inc., San Diego, CA, USA) or COBAS AMPLICOR (Roche Diagnostics, Tokyo, Japan) systems or by DNA–DNA hybridization assays (Kyokuto Pharmaceutical Industrial, Tokyo, Japan).

### 2.6. Statistical Analysis

All statistical analyses were performed using GraphPad Prism version 7 (GraphPad Software, San Diego, CA, USA) and JMP Pro 13 (SAS Institute, Gary, NC, USA). Continuous variables were reported as median and interquartile range. The data were compared using the *t*-test for continuous variables such as age, and the χ^2^ test for categorical variables. When any cells had an expected count of less than 5, Fisher’s exact test was used instead of the χ^2^ test. Factors showing probability values <0.10 in univariate analyses were evaluated by multivariate cox and logistic regression analysis. Probability values < 0.05 were regarded as statistically significant.

## 3. Results

### 3.1. Baseline Characteristics and Analysis of Prognostic Impacts of Aspergillus Isolation

During the study period, 1219 NTM-PD patients were identified. Two patients who developed SA was excluded. Overall, 409 patients with NTM-PD who underwent fungal examinations including fungal cultures of respiratory specimen at least once were study population of this study. The median observation period was 85 months after diagnosis. The baseline clinical characteristics of all patients are presented in Table 1. Of these 409 patients, 264 (64.5%) were women (mean age, 62 years) and 325 (79.5%) were *Mycobacterium avium* complex pulmonary disease. During the observation period, *Aspergillus* species were isolated from 79 patients (79/409, 19.3%). The most frequently detected species were *A. fumigatus* (56/79, 70.9%) and *A. niger* (18/79, 22.8%).

**Figure 1:**
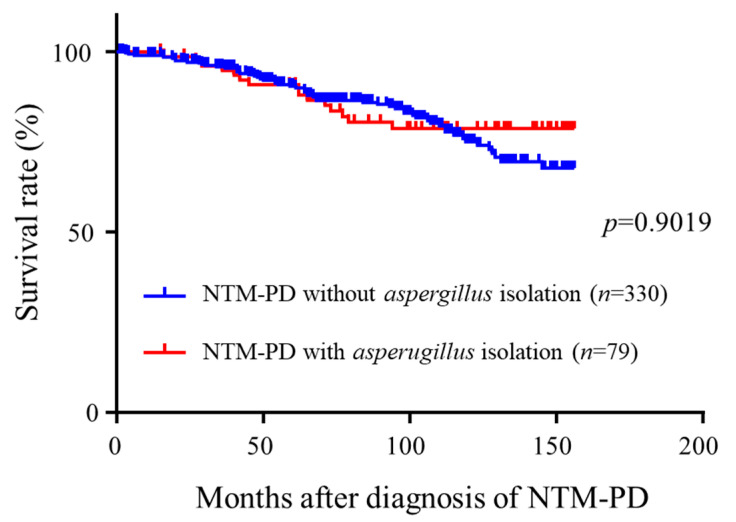
Comparison of survival curves of NTM-PD patients with and without *asperugillus* isolation. NTM-PD, non-tuberculous mycobacterium pulmonary disease.

We first analyzed the prognostic factors of NTM-PD patients. Death from any cause occurred in 73 patients. The causes of death included pneumonia (*n* = 13, 17.8%), progression of NTM-PD (*n* = 35, 47.9%), CPA (*n* = 3, 4.1%), lung cancer (*n* = 9, 12.3%), other pulmonary diseases (*n* = 8, 11.0%), non-pulmonary diseases (*n* = 4, 5.5%), and unknown causes (*n* = 1, 1.4%). Univariate and multivariate cox regression analysis revealed that age (*p* < 0.0001), body mass index (BMI) (*p* < 0.0001), C-reactive protein (CRP) (*p* = 0.0077), cavity (*p* = 0.0036), and acid fast bacilli (AFB) stain positivity (*p* = 0.0001) were significantly and independently associated with mortality. *Aspergillus* isolation was not associated with mortality (Table 1). We further analyzed the survival curves of NTM-PD patients (Figure 1). During the observation period, 13 (16.5%) of 79 patients with Aspergillus isolation and 60 (17.9%) of 336 patients without Aspergillus isolation died (*p* =0.9016). Survival curves of *Aspergillus* isolated patients were comparable to NTM-PD patients without *Aspergillus* isolation (*p* = 0.9019), suggesting that classification of patients simply stratified by the presence or absence of *Aspergillus* isolation would not have predictive values in NTM-PD patients.

### 3.2. Risk Factors for Aspergillus Isolation

We next analyzed the risk factors for aspergillus isolation. Univariate logistic regression analysis revealed that systemic steroid use (*p* = 0.0104), higher CRP (*p* = 0.0087), and AFB stain positivity (*p* = 0.0033) were significantly associated with *Aspergillus* isolation. Multivariate logistic regression analysis revealed that higher CRP (*p* = 0.0213), and AFB stain positivity (*p* = 0.0101) were independently associated with *Aspergillus* isolation (Table 2). Different mycobacterial species were not associated with *Aspergillus* isolation.

### 3.3. Risk Factors for CPA Development

**Figure 2:**
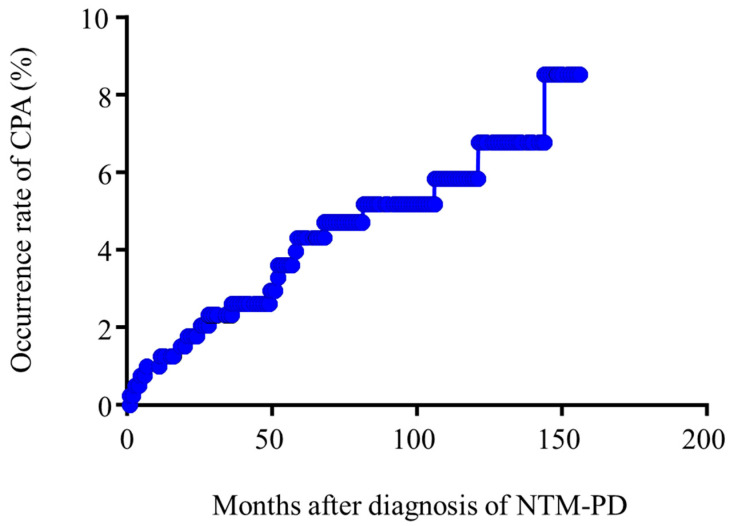
Cumulative occurrence rate of chronic pulmonary aspergillosis (CPA) in NTM-PD patients. NTM-PD, non-tuberculous mycobacterial pulmonary disease; CPA, chronic pulmonary aspergillosis.

**Figure 3:**
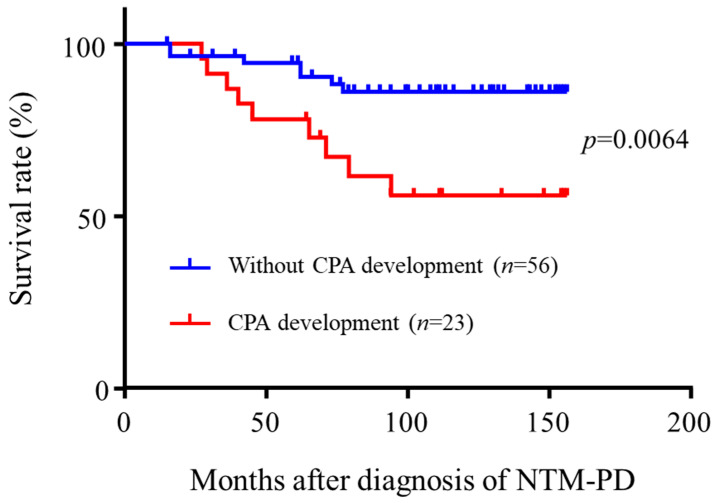
Survival curves of NTM-PD patients with *aspergillus* isolation. NTM-PD, non-tuberculous mycobacterial pulmonary disease; CPA, chronic pulmonary aspergillosis.

During the observation period, 23 (29.1% of *Aspergillus* isolated patients) developed CPA. Median time for the development of CPA from the diagnosis of NTM-PD was 2.31 years (Interquartile range; 0.39–4.82) (Figure 2). Of the 23 patients who developed CPA, fourteen (60.9%) were treated with oral itraconazole, eight (34.8%) were treated with oral voriconazole, and one (4.3%) were treated with intravenous caspofungin due to the drug interactions with rifampicin. Survival curves for patients with CPA diagnosis were significantly and strikingly different from those without (*p* = 0.0064), suggesting that CPA development severely affects clinical outcome (Figure 3). Univariate logistic regression analysis revealed that Old tuberculosis (Tb) (*p* = 0.0409), use of systemic steroids (*p* = 0.0069), non-nodular bronchiectatic form of the disease (*p* = 0.0045), cavity (*p* = 0.0005), and AFB stain positivity (*p* = 0.0021) were significantly associated with CPA development in NTM-PD patients. (Table 3). Multivariate logistic regression analysis revealed that use of systemic steroids (*p* = 0.0189) and cavity (*p* = 0.0207) were independently associated with CPA development in NTM-PD patients.

### 3.4. Analysis of Risk Factors for Mortality in Aspergillus Isolated NTM-PD Patients

Although aspergillus isolation alone was not associated with poor clinical outcomes. CPA occurs distinct sub-populations in association with immune suppressive state and cavitary disease, and development of CPA in these patients led to strikingly poor survival outcomes. These results suggested that aspergillus isolation in NTM-PD patients could be divided into two distinct conditions; colonization and infection. Furthermore, survival curve analysis and prognostic factor analysis in *aspergillus* isolated NTM-PD patients suggested that colonization would be the major form of these conditions. With these results, we next analyzed the factors that associated with mortality in order to explore the differences between colonization, the prognosis of which is comparable to non-*Aspergillus* isolated patients, and infection, which leads to poor clinical outcomes. Univariate cox regression analysis revealed that male sex (*p* = 0.041), cavity (*p* = 0.0007), and AFB stain positivity (0.0006) were significantly associated with mortality in *Aspergillus* isolated NTM-PD patients (Table 4). Multivariate cox regression analysis revealed that male sex (*p* = 0.0345), cavity (0.0056), and AFB stain positivity (*p* = 0.0051) were independent risk factors for mortality in *Aspergillus* isolated NTM-PD patients. Although not statistically different, mortality of patients with non-NB form of the disease and *A.fumigatus* isolation were higher in *Aspergillus* isolated NTM-PD patients. *A.fumigatus* isolation in male patients with active cavitary NTM-PD would have highest risk of progression to *Aspergillus* infection.

## 4. Discussion

This is the first and largest study to assess the impacts and prevalence of *Aspergillus* isolation and CPA in NTM-PD patients using definite diagnostic criteria for CPA. In this study, we showed that isolation of *aspergillus* species (79/409, 19.3%) and development of CPA (23/409, 5.3%) were prevalent among NTM-PD patients. We also showed that isolation of *Aspergillus* species alone was not associated with poor prognosis in NTM-PD patients, and more than half of patients with *Aspergillus* isolation did not develop CPA. Furthermore, NTM-PD patients from whom *Aspergillus* species were isolated could be clearly divided into two distinct subgroups according to their immunological responses to *Aspergillus* species (those with *Aspergillus* colonization and those with infection such as CPA). Chronic inflammatory status, systemic steroid use, and cavitary disease are risk factors for NTM-PD patients for isolation of *Aspergillus* species and progression to CPA. In addition, cavitary non-NB form of the disease, and detection of *A. fumigatus* were possible risk factors for the progression from *Aspergillus* colonization to infection.

CPA is a fungal chronic progressive infection caused by *Aspergillus* species, which are ubiquitous fungi. Lots of underlying conditions that affects and weakens host immune responses have been reported to be associated with CPA development. Previous studies have consistently reported that preexisting lung disease such as NTM infection is an important underlying condition. In this study, we evaluated the impact of *Aspergillus* isolation and development of CPA in NTM-PD patients on outcomes. During the study period, *Aspergillus* species were isolated in 19.3% (79/409) of NTM-PD patients, 5.6% (23/409) developed CPA, which coincides with the previous studies that reported the prevalence of CPA development in NTM-PD patients to be 3.9% to 16.7% [9,10,11]. These relatively high incidences of *aspergillus* isolation and CPA development in NTM-PD patients suggest that NTM-PD patients were more likely to develop *Aspergillus* colonization and infection than other chronic pulmonary disease. Indeed, in countries with a low prevalence of tuberculosis, NTM-PD was identified as the second leading cause of CPA (14.9%) following pulmonary tuberculosis (15.3%) [12]. These results prompted us to anticipate the close association between NTM and *Aspergillus*.

Our results suggested that isolation of *Aspergillus* species in NTM-PD patients might be mostly the reflection of colonization. *Aspergillus* colonization is partly a consequence of airway clearance defects associated with bronchiectatic disease, and the prognosis in these patients was comparable to NTM-PD without *Aspergillus* isolation. Airway clearance defects are known to be associated with development and progression of NTM-PD as well as fungal pulmonary disease in bronchiectatic patients [4]. Hence, *Aspergillus* colonized NTM-PD patients would benefit from airway clearance therapies in addition to optimal combinations of antibiotic therapies, and antifungal drug treatments are not supposed to be necessary for *Aspergillus* colonization. The drug–drug interactions of azoles with RFP are well known, and unnecessary administration of these antifungal drugs to *Aspergillus* colonized NTM-PD patients makes it difficult to select suitable therapeutic drugs for NTM-PD.

Prognostic factors for NTM-PD have been identified in many retrospective studies [3,13,14,15]. In our study, higher age (*p* < 0.0001), low BMI (*p* < 0.0001), high CRP levels (*p* = 0.0077), cavitation (*p* = 0.0036), and AFB stain positivity (*p* = 0.0001) were significantly and independently associated with mortality. On the other hands, mycobacterial species and *Aspergillus* isolation were not associated with mortality.

We also showed that NTM-PD with CPA development was associated with extremely poor prognosis, and that cavitation was significantly associated with CPA, and previous studies by Ishikawa et al. and Fujiuchi et al. also found the same results [10,11]. Cavity formation during mycobacterial infection is associated with massive necrosis and loss of normal architecture in peripheral areas of the lung, and is induced by macrophages and activated T cells in response to infection as well as by delayed-type hypersensitivity to mycobacterial antigens [16,17]. This may also be the case for NTM-PD. *Aspergillus* can also lead to host hypersensitivity, which may contribute to the destruction of lung tissue in combination with inflammatory responses against the organism itself [11]. Therefore, co-infection with NTM and *Aspergillus* species may exaggerate lung tissue destruction and subsequent respiratory failure.

The association between different NTM species and incidence of CPA has not been fully elucidated. Regarding the causative NTM species of CPA developed patients, Fujiuchi et al. reported that M. kansasii was more likely to be complicated by CPA, and Jhun et al. reported that *M.abscessus* was more likely to be complicated by CPA than *Mycobacterium avium* complex [2,12]. However, there was no tendency of causative mycobacterium in our study, and *Mycobacterium avium* complex is the most common NTM species that causes CPA in this study.

Furthermore, prognostic factor analysis by univariate and multivariable analysis revealed that use of systemic steroids was significantly and independently associated with the development of CPA. Previous study by Garnacho-Montero et al. showed that use of steroids was an independent risk factor for colonization of *Aspergillus* species [18]. Jhun et al. also reported that use of systemic steroids to be an independent risk factor for the development of CPA in NTM-PD patients [2]. Immunosuppressive effects of steroids lead to weakened host defense that might be one possible cause of the development of CPA.

As above, identification and proper management of CPA in NTM-PD patients are extremely important. However, our understandings of incidence and prevalence of CPA in NTM-PD patients are still unsatisfactory, which have made it difficult for the establishment of screening strategies for CPA in NTM-PD patients. In our study, 5.3 % of NTM-PD patients developed CPA, and the median time to diagnosis of NTM-PD was 2.31 years. These were consistent with the previous reports by Takeda et al. [19], and Jhun et al. [2]. Based on these results, NTM-PD patients who have potential risk factors for *Aspergillus* infection, performing the serum *Aspergillus* precipitin test and fungal cultures at least once a year would help early diagnosis and proper management of CPA in NTM-PD patients.

The current study had several limitations. First, this study was confined to a single referral center for respiratory diseases. Typical high-risk patients for *Aspergillus* infection such as patients with immunodeficient disorders, blood tumor patients, and organ transplant patients are not included in our study. Second, we could not exclude potential confounding factors (e.g., microbiological and other laboratory features) because of the retrospective nature of our study. Third, we excluded patients who had not been performed fungal culture testing of respiratory specimens. We excluded these patients to reduce potential biases, because *Aspergillus* species will never be detected in patients without performing fungal cultures. Although, most of the excluded patients are judged as unlikely to have aspergillosis by attending physicians or had less expectoration of sputum, we cannot exclude the possibilities that the results would be affected to some extent by this exclusion. Fourth, although clinical CPA diagnosis can be made by either positive serology or positive culture results in patients with compatible clinical and radiological symptoms, serologically positive patients without aspergillus isolation were not included in CPA in this study. NTM-PD patients who underwent fungal cultures were the patient cohort of this study. If serologically positive patients without aspergillus isolation were included in CPA, these patients should be compared to all NTM-PD patients irrespective of performing fungal cultures, and it may lead to the inconsistency with the patient cohort of those who have been tested for fungi. Therefore, in this study, we regarded patients as confirmed CPA cases only if all of the clinical, radiological, serological, and culture tests were positive. Finally, we did not evaluate factors affecting decision-making by pulmonary physicians regarding the timing of regular screening tests for fungal infections.

## 5. Conclusions

In conclusion, *Aspergillus*-related conditions in NTM-PD can be classified as totally different subgroups: *Aspergillus* colonization and chronic infection such as CPA development. *Aspergillus* colonization would be a consequence of airway clearance defects, and the prognosis is comparable to NTM-PD without *Aspergillus* related conditions. Considering the high mortality rate of CPA in NTM-PD, early diagnosis and treatment are essential to improve outcomes for NTM-PD patients.

## Figures and Tables

**Table 1:** Base line characteristics and prognostic factors of non-tuberculous mycobacteria pulmonary disease (NTM-PD) patients.

				Univariate Analysis		Multivariate Analysis	
	Total NTM-PD (*n* = 409)	Sensored (*n* = 336)	Death (*n* = 73)	*p*-Value	HR (95%CI)	*p*-Value	Adjusted HR (95% CI)
Characteristic							
Sex, male	145 (35.5)	112 (33.3)	33 (45.2)	0.0169 *	1.77 (1.11–2.81)	0.0487 *	1.83 (1.00–3.33)
Age, years	62.41 (±11.06)	61.78 (±11.02)	65.29 (±10.86)	<0.0001 *	1.05 (1.03–1.08)	<0.0001 *	1.06 (1.03–1.09)
BMI	18.98 (±2.949)	19.28 (±2.76)	17.75 (±3.371)	<0.0001 *	0.03 (0.006–0.17)	<0.0001 *	1.0 (1.03–1.21)
Underlying disease							
COPD	43 (10.5)	32 (9.5)	11 (15.1)	0.2033	1.55 (0.77–2.82)		
Old Tb	17 (4.2)	13 (3.9)	4 (5.5)	0.8891	1.08 (0.33–2.60)		
DM	64 (15.6)	46 (13.7)	18 (24.7)	0.0221 *	1.94 (1.11–3.23)	0.3376	1.37 (0.70–2.52)
Use of systemic steroid	49 (12.0)	31 (9.2)	18 (24.7)	0.0054 *	2.30 (1.30–3.88)	0.2926	1.40 (0.73–2.54)
CRP	1.69 (±2.804)	1.344 (±2.641)	2.884 (±3.035)	0.0001 *	1.14 (1.07–1.19)	0.0077 *	1.12 (1.03–1.21)
NB form	317 (77.5)	267 (79.5)	50 (68.5)	0.0092 *	0.50 (0.31–0.84)	0.3868	0.75 (0.39–1.43)
Cavity	149 (36.4)	103 (30.7)	46 (63.0)	<0.0001 *	4.07 (2.54–6.67)	0.0036 *	2.38 (1.33–4.38)
Species							
MAC	325 (79.5)	270 (80.4)	55 (75.3)	0.6984	0.89 (0.50–1.69)		
*M. abscessus*	32 (7.8)	26 (7.7)	6 (8.2)				
*M. kansasii*	26 (6.4)	22 (6.5)	4 (5.5)				
Others	24 (5.9)	18 (5.4)	6 (8.2)				
AFB stain positive	111 (27.1)	72 (21.4)	39 (53.4)	<0.0001 *	4.11 (2.59–6.55)	0.0001 *	2.87 (1.68–4.95)
*Aspergillus* isolation	79 (19.3)	64 (19.0)	13 (17.8)	0.9016	0.97 (0.54–1.64)		
Isolated *Aspergillus* species							
*A. fumigatus*	56 (13.7)	42 (12.5)	14 (19.2)				
*A. niger*	18 (4.4)	17 (5.1)	1 (1.4)				
Others	5 (1.2)	5 (1.5)	0 (0.0)				

NTM, non-tuberculous mycobacterium; MAC, *Mycobacterium avium* complex; HR, hazard ratio; CI, confidence interval; BMI, body mass index; COPD, chronic obstructive pulmonary disease; Tb, tuberculosis; DM, diabetes mellitus; RA, rheumatoid arthritis; CRP, C-reactive protein; NB, nodular bronchiectasis; AFB, acid-fast bacilli; CPA, chronic pulmonary aspergillosis. Data represent *n* (%) or mean (±standard deviation). * *p* < 0.05 by univariate and multivariate cox regression analysis.

**Table 2:** Risk factors for *Aspergillus* isolation in NTM-PD patients.

			Univariate Analysis		Multivariate Analysis	
	Without Aspergillus Isolation (*n* = 330)	With Aspergillus Isolation (*n* = 79)	*p*-Value	OR (95%CI)	*p*-Value	Adjusted OR (95%CI)
Characteristic						
Sex, male	110 (33.3)	35 (44.3)	0.0684	1.59 (0.97–2.62)		
Age	62.68 (±11.3)	61.25 (±9.8)	0.3188	0.99 (0.97–1.01)		
BMI	19.02 (±2.9)	18.81 (±3.1)	0.5924	0.98 (0.89–1.07)		
Underlying disease						
COPD	33 (10.0)	10 (12.7)	0.4900	1.30 (0.61–2.77)		
Old Tb	13 (3.9)	4 (5.1)	0.6539	1.30 (0.41–4.10)		
DM	52 (15.8)	12 (15.2)	0.9007	0.96 (0.48–1.89)		
Use of systemic steroid	33 (10.0)	16 (20.3)	0.0104 *	2.37 (1.22–4.57)	0.3053	1.48 (0.70–3.15)
CRP	1.45 (±2.4)	2.57 (±3.8)	0.0087 *	1.13 (1.03–1.23)	0.0213 *	1.11 (1.02–1.22)
NB form	259 (78.5)	58 (73.4)	0.3336	0.76 (0.43–1.33)		
Cavity	119 (36.1)	30 (38.0)	0.7896	1.07 (0.65–1.78)		
Species						
MAC	260 (78.8)	65 (82.3)	0.6897	1.14 (0.60–2.16)		
*M. abscessus*	26 (7.9)	6 (7.6)				
*M. kansasii*	21 (6.4)	5 (6.3)				
Others	21 (6.4)	3 (3.8)				
AFB stain positive	79 (23.9)	32 (40.5)	0.0033 *	2.16 (1.29–3.62)	0.0101 *	2.14 (1.20–3.83)

NTM, non-tuberculous mycobacterium; MAC, *Mycobacterium avium* complex; OR, odds ratio; CI, confidence interval; BMI, body mass index; COPD, chronic obstructive pulmonary disease; Tb, tuberculosis; DM, diabetes mellitus; RA, rheumatoid arthritis; CRP, C-reactive protein; NB, nodular bronchiectasis; AFB, acid-fast bacilli; CPA, chronic pulmonary aspergillosis. Data represent *n* (%) or mean (±standard deviation). * *p* < 0.05 by univariate and multivariate logistic regression analysis.

**Table 3:** Risk factors for CPA development in NTM-PD patients.

	Univariate Analysis		Multivariate Analysis	
	*p*-Value	OR (95%CI)	*p*-Value	Adjusted OR (95%CI)
Characteristic				
Sex, male	0.4096	1.43 (0.61–3.35)		
Age	0.5835	0.96 (0.93–1.00)		
BMI	0.3792	0.94 (0.81–1.08)		
Underlying disease				
COPD	0.6846	1.30 (0.37–4.56)		
Old Tb	0.0409 *	0.29 (1.06–15.00)	0.0613	4.10 (0.94–17.99)
DM	0.1633	2.00 (0.76–5.27)		
Use of systemic steroid	0.0069 *	3.68 (0.12–0.68)	0.0189 *	3.31 (1.22–9.01)
CRP	0.1228	1.10 (0.98–1.24)		
NB form	0.0045 *	0.29 (0.12–0.68)	0.1804	0.52 (0.20–1.35)
Cavity	0.0005 *	5.39 (2.08–13.99)	0.0207 *	3.48 (1.21–9.98)
Species				
MAC	0.1707	0.52 (0.21–1.32)		
AFB stain positive	0.0021 *	3.82 (1.62–8.99)	0.1168	2.11 (0.83–5.37)

NTM, non-tuberculous mycobacterium; MAC, *Mycobacterium avium* complex; OR, odds ratio; CI, confidence interval; BMI, body mass index; COPD, chronic obstructive pulmonary disease; Tb, tuberculosis; DM, diabetes mellitus; RA, rheumatoid arthritis; CRP, C-reactive protein; NB, nodular bronchiectasis; AFB, acid-fast bacilli; CPA, chronic pulmonary aspergillosis. Data represent *n* (%) or mean (±standard deviation). * *p* < 0.05 by univariate and multivariate logistic regression analysis.

**Table 4:** Risk factors for mortality in aspergillus isolated NTM-PD patients.

	Univariate Analysis		Multivariate Analysis	
	*p*-Value	HR (95%CI)	*p*-Value	Adjusted HR (95%CI)
Characteristic				
Sex, male	0.041 *	2.82 (1.04–8.33)	0.0345	3.08 (1.09–9.61)
Age,	0.1001	1.05 (0.99–1.12)		
BMI	0.1115	0.86 (0.71–1.03)		
Underlying disease				
COPD	0.9468	1.05 (0.17–3.77)		
Old Tb	0.8338	1.25 (0.07–6.18)		
DM	0.2909	1.91 (0.53–5.48)		
Use of systemic steroid	0.3113	1.77 (0.56–4.86)		
CRP	0.2518	1.08 (0.94–1.21)		
NB form	0.11	0.44 (0.16–1.22)		
Cavity	0.0007 *	5.96 (2.07–21.3)	0.0056 *	4.53 (1.53–16.59)
Species				
MAC	0.7789	0.83 (0.26–3.64)		
AFB stain positive	0.0006 *	6.16 (2.14–22.1)	0.0051	4.63 (1.56–16.96)
Isolated *Aspergillus* species				
*A. fumigatus*	0.0529	3.54 (0.99–22.54)	0.1548	2.67 (0.72–17.36)

NTM, non-tuberculous mycobacterium; MAC, *Mycobacterium avium* complex; HR, hazard ratio; CI, confidence interval; BMI, body mass index; COPD, chronic obstructive pulmonary disease; Tb, tuberculosis; DM, diabetes mellitus; CRP, C-reactive protein; NB, nodular bronchiectasis; AFB, acid-fast bacilli; CPA, chronic pulmonary aspergillosis. Data represent n (%) or mean (±standard deviation). * *p* < 0.05 by univariate and multivariate cox hazard analysis.

## Data Availability

The datasets supporting the conclusions of this article are included within the article.

## References

[1] Furuuchi K., Ito A., Hashimoto T., Kumagai S., Ishida T. (2018). Clinical significance of *Aspergillus* species isolated from respiratory specimens in patients with *Mycobacterium avium* complex lung disease. Eur. J. Clin. Microbiol. Infect. Dis. Off. Publ. Eur. Soc. Clin. Microbiol..

[2] Jhun B.W., Jung W.J., Hwang N.Y., Park H.Y., Jeon K., Kang E.S., Koh W.J. (2017). Risk factors for the development of chronic pulmonary aspergillosis in patients with nontuberculous mycobacterial lung disease. PLoS ONE.

[3] Fukushima K., Kitada S., Abe Y., Yamamoto Y., Matsuki T., Kagawa H., Oshitani Y., Tsujino K., Yoshimura K., Miki M. (2020). Long-Term Treatment Outcome of Progressive *Mycobacterium avium* Complex Pulmonary Disease. J. Clin. Med..

[4] Phoompoung P., Chayakulkeeree M. (2020). Chronic Pulmonary Aspergillosis Following Nontuberculous Mycobacterial Infections: An Emerging Disease. J. Fungi.

[5] Denning D.W., Cadranel J., Beigelman-Aubry C., Ader F., Chakrabarti A., Blot S., Ullmann A.J., Dimopoulos G., Lange C. (2016). Chronic pulmonary aspergillosis: Rationale and clinical guidelines for diagnosis and management. Eur. Respir. J..

[6] Godet C., Philippe B., Laurent F., Cadranel J. (2014). Chronic pulmonary aspergillosis: An update on diagnosis and treatment. Respir. Int. Rev. Thorac. Dis..

[7] Delliere S., Angebault C., Fihman V., Foulet F., Lepeule R., Maitre B., Schlemmer F., Botterel F. (2019). Concomitant Presence of *Aspergillus* Species and Mycobacterium Species in the Respiratory Tract of Patients: Underestimated Co-occurrence?. Front. Microbiol..

[8] Griffith D.E., Aksamit T., Brown-Elliott B.A., Catanzaro A., Daley C., Gordin F., Holland S.M., Horsburgh R., Huitt G., Iademarco M.F. (2007). An official ATS/IDSA statement: Diagnosis, treatment, and prevention of nontuberculous mycobacterial diseases. Am. J. Respir. Crit. Care Med..

[9] Zoumot Z., Boutou A.K., Gill S.S., van Zeller M., Hansell D.M., Wells A.U., Wilson R., Loebinger M.R. (2014). *Mycobacterium avium* complex infection in non-cystic fibrosis bronchiectasis. Respirology.

[10] Ishikawa S., Yano S., Kadowaki T., Wakabayashi K., Kimura M., Kobayashi K., Ikeda T. (2011). Clinical analysis of non-tuberculous mycobacteriosis cases complicated with pulmonary aspergillosis. Kekkaku.

[11] Fujiuchi S., Sakunami M., Yamamoto Y., Takeda A., Nishigaki Y., Fujita Y., Yamazaki Y., Fujikane T. (2008). Analysis of chronic necrotizing pulmonary aspergillosis (CNPA) cases complicated with non-tuberculous mycobacteriosis (NTM). Kekkaku.

[12] Smith N.L., Denning D.W. (2011). Underlying conditions in chronic pulmonary aspergillosis including simple aspergilloma. Eur. Respir. J..

[13] Hayashi M., Takayanagi N., Kanauchi T., Miyahara Y., Yanagisawa T., Sugita Y. (2012). Prognostic factors of 634 HIV-negative patients with *Mycobacterium avium* complex lung disease. Am. J. Respir. Crit. Care Med..

[14] Fukushima K., Miki M., Matsumoto Y., Uda E., Yamamoto Y., Kogita Y., Kagawa Y., Matsuki T., Kagawa H., Oshitani Y. (2020). The impact of adjuvant surgical treatment of nontuberculous mycobacterial pulmonary disease on prognosis and outcome. Respir. Res..

[15] Fukushima K., Kitada S., Komukai S., Kuge T., Matsuki T., Kagawa H., Tsujino K., Miki M., Miki K., Kida H. (2021). First line treatment selection modifies disease course and long-term clinical outcomes in *Mycobacterium avium* complex pulmonary disease. Sci. Rep..

[16] Yamamura Y., Maeda H., Ogawa Y., Hashimoto T. (1986). Experimental pulmonary cavity formation by mycobacterial components and synthetic adjuvants. Microbiol. Immunol..

[17] Kaplan G., Post F.A., Moreira A.L., Wainwright H., Kreiswirth B.N., Tanverdi M., Mathema B., Ramaswamy S.V., Walther G., Steyn L.M. (2003). Mycobacterium tuberculosis growth at the cavity surface: A microenvironment with failed immunity. Infect. Immunity.

[18] Garnacho-Montero J., Amaya-Villar R., Ortiz-Leyba C., Leon C., Alvarez-Lerma F., Nolla-Salas J., Iruretagoyena J.R., Barcenilla F. (2005). Isolation of *Aspergillus* spp. from the respiratory tract in critically ill patients: Risk factors, clinical presentation and outcome. Crit. Care.

[19] Takeda K., Imamura Y., Takazono T., Yoshida M., Ide S., Hirano K., Tashiro M., Saijo T., Kosai K., Morinaga Y. (2016). The risk factors for developing of chronic pulmonary aspergillosis in nontuberculous mycobacteria patients and clinical characteristics and outcomes in chronic pulmonary aspergillosis patients coinfected with nontuberculous mycobacteria. Med. Mycol..

